# Case Report: *Rhizopus arrhizus* Rhino-Orbital-Cerebral Mycosis and Lethal Midline Granuloma: Another Fungal Etiological Agent

**DOI:** 10.3389/fmed.2021.578684

**Published:** 2021-06-02

**Authors:** Dong Ming Li, Li De Lun, Jie Ge, Gong Jie Zhang, Xin Lun Li, G. Sybren de Hoog

**Affiliations:** ^1^Division of Dermatology & Mycological Lab, Peking University Third Hospital, Beijing, China; ^2^Division of Nephrology, Air Force General Hospital, Beijing, China; ^3^Center of Expertise in Mycology of Radboud University Medical Center, Canisius Wilhelmina Hospital, Nijmegen, Netherlands

**Keywords:** midline face destruction, *Rhizopus arrhizus*, rhino-orbital-cerebral mycosis, NK/T cells, *Rhizopus oryzae*, lethal midline granuloma

## Abstract

**Objective:** Both rhino-orbital-cerebral mycosis and lethal midline granuloma (LMG) may result in midline destruction. LMG has now been generally considered as a natural killer/T cell lymphoma, nasal type (ENKTL-NT) with an association of EBV. Fungi have been detected from the diseased tissues now and then but are often considered as lymphoma-associated infections. We previously reported an ENKTL-NT case with *Mucor irregularis*, which played a causal role in the disease and was involved in the overexpression of Ki67 and CD56 in the mouse experiment. The present study describes a chronic *Rhizopus arrhizus* infection with immunological parameters that are closely similar to LMG. We aim to explore the relationship of another Mucorales fungus, *R. arrhizus*, and LMG in a patient and in mice.

**Methods:** Case study and mouse infection modules were designed for our observation. A 35-year-old man with midline face ulcers which was clinically suspected as LMG was selected. Biopsy specimens were sent for lymphoma diagnosis and microbiological detection. The isolated fungus was tested in an ICR mouse model for mycological and histological analyses.

**Results:** Five tissue samples yielded *Rhizopus arrhizus*. In the pathology, characteristic inflammation, necrosis, and granulation with thin-walled hyphae are observed. Immunohistochemistry showed NK/T cell infiltration (CD3+, CD8+, TIA1+, GZMB+, PRF+, individual CD56+) with hyperplasia (Ki67+) and angioinvasion. The patient recovered completely with amphotericin B. In the murine experiment, *R. arrhizus* caused angioinvasion with NK/T cell infiltration (CD3+, CD56+, TIA1+, GZMB +, PRF+) with proliferation (Ki67+) and was re-isolated from the infected host.

**Conclusions:** We here describe a mid-face destruction patient, which was diagnosed by the top pathologists in China according to the current criteria of NK/T cell lymphoma, with a negative result for EBV and positive result for *R. arrhizus*. With a then developed mouse experiment, the *R. arrhizus* in the diseased lesions was responsible for the NK/T cell infiltration (CD3+, CD8+, CD56+, TIA1+, GZMB+, PRF+), proliferation (Ki67+), and angioinvasion, suggesting another fungal etiological agent for LMG, which could be eradicated with amphotericin B.

**Limitations:** The sample size is not sufficient for statistical analysis. However, our findings are suggestive for the role fungus plays in LMG.

## Introduction

Lethal midline granuloma (LMG) is a clinical-pathological entity characterized by progressive midline face ulceration, granulation, and destruction, with a high mortality rate (1, 2). The disorder was first described as a malignant granuloma for the rapid destruction of the face and nose by Mcbride in 1897 (3, 4). With a variety of descriptive names such as “non-healing granuloma,” “granuloma gangraenescens,” “malignant granuloma,” “idiopathic midline destructive disease,” and “lethal midline granuloma” (5–8), it is now known as the “extra nodule natural killer NK/T cell lymphoma, nasal type (ENKTL-NT),” because natural killer cells, although on a lesser extent T cells, are often detected along with cytotoxic granule associated proteins (9–11). However, diagnosis and treatment of LMG has remained challenging; in some cases, the condition of patients worsened due to anti-cancer therapy (2, 12–16) in the early or later stages (17), or achieved resolution with antifungal therapy alone or in combination with other medication (18–21), as what can also be obtained with rhino-orbital-cerebral mucormycosis (ROCM) (22).

The role of fungi isolated from clinical sites may be unclear. However, the clinical similarity of mucoralean fungal infections may lead to an inappropriate therapy. Mixed cases have been reported where the fungal infection was cured but the anticancer therapy was continued, leading to adverse results (2, 23). Some LMG patients received chemotherapy, but *post-mortem*, the cause of their disorder was shown to be fungal (24, 25). In patients primarily diagnosed with ROCM, the detection of NK cells and/or T cells in the tissue led to a change of diagnosis to from ROCM to LMG, with a fatal outcome (1, 26). In another paper, two ROCM cases were reported with NK/T cells and angiocentric characteristics. Diagnosis for one patient was changed from ENKTL-NT to ROCM as fungal mycelium was seen in the tissue, while the diagnosis for the other was from ROCM to NK/T cell lymphoma because the NK/T cells were seen in the tissue (27). These cases suggest the ambiguity in understanding the role of fungi in the development of midline ulcers (27, 28).

We previously identified a chronic infection by *Mucor irregularis* as the fungal cause of a case resembling LMG (28, 29). Since other mucoralean fungi tend to cause acute infections in susceptible patient populations, it seemed unlikely that other Mucorales fungi could be associated with the disorder. Here, we report a case of a Chinese male who presented with midline facial destruction which was diagnosed as LMG, but the etiologic agent was *Rhizopus arrhizus*, another member of Mucorales, confirmed in histopathology and isolation. Replication of NK/T cells, angioinvasion, and hyperplasia were observed and reproduced in a mouse model.

### Case Presentation

A 35-year-old Chinese male with diabetes mellitus presented to Peking University Third Hospital (Beijing, China) on November 14, 2011, with progressive swelling, ulceration, and destruction of the face. The lesions had begun 2 months earlier as infiltrated rhino facial and maxillofacial erythema, with nasal obstruction, discharge, rhinorrhea, and epistaxis, which rapidly increased in size and ulcerated. The patient was initially diagnosed, in the local hospital, with periodontitis and the treatment with tooth extraction was ineffective. Next, the lesion was diagnosed with skin and subcutaneous infection in the rhino face and treatment with antibiotics was administered, but the necrotic ulcer progressively enlarged. Subsequent clinical diagnosis was made of rhino facial mycosis and the patient was treated with itraconazole, but the ulcer still progressed with the disease eroding through his hard palate, sinuses, nose, and face, resulting in destructive changes in the maxilla and mandible, with systemic manifestations of fever, weight loss, and hepatosplenomegaly. As the ulceration progressed, he was suspected as having LMG and was therefore transferred 2 weeks after to the university clinic for further diagnosis and treatment.

**Figure 1:**
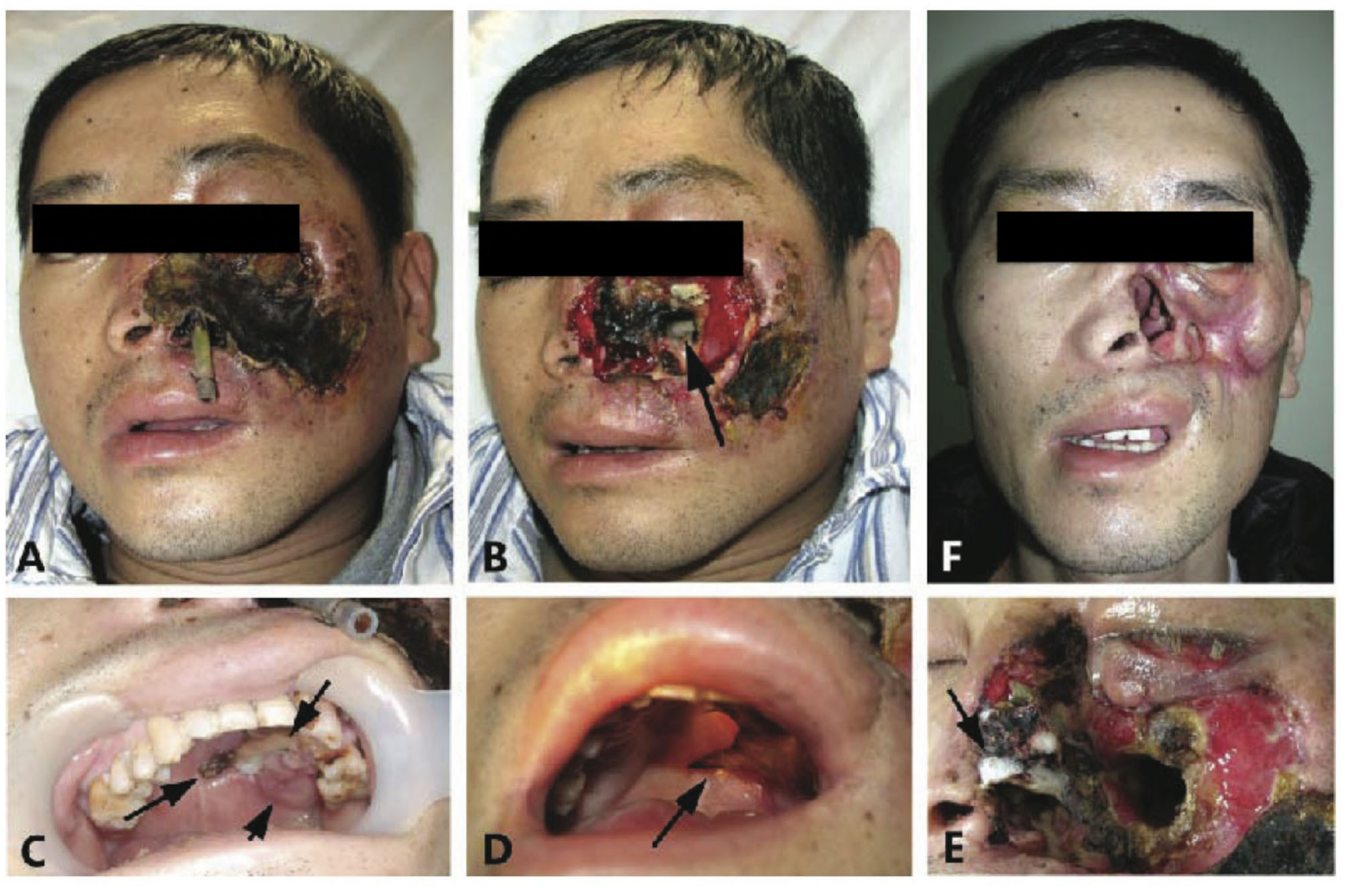
“*Rhizopus arrhizus* associated lethal midline granuloma/rhino-orbital cerebral mycosis.” Destruction of the sinus, nose, face, palate, and orbit; defects of the left sided external nose, soft tissues, and the front wall of the maxillary sinus; and yellow pus was seen within the left maxillary sinus. **(A,B)** Show patient onset before and after debridement with biopsies. **(C)** Reveals the patches dark discoloration (short arrow), light discoloration (medium arrow), and perforation (long arrow) of the hard palate that represent inflammation, ischemia, and necrosis, respectively. **(D)** Shows widespread destruction in the left side of the palate (arrow). **(E)** Reveals pale gray wool-like mycelium grown on the necrotic nose tissue (arrow). **(F)** Shows complete remission with scars 4 months after antifungal therapy.

On examination, his left half central face was swollen, ulcerated, and destructed, and the nose, lower eyelid, chin, and upper lip were all necrotic. Black eschar covered the left side of the face with an underlying grayish pus (Figure 1A). The anterior wall of the left maxilla was perforated with a green-yellowish pus seen at the bottom of the maxilla (Figure 1B). The hard palate had patches of black, pale discoloration showed perforation (Figure 1C), and soon proceeded with complete destruction (Figure 1D). Pale gray, wool-like mycelium was observed on the necrotic tissue of the nose (Figure 1E). The patient had a normal temperature, pulse, respiration, and blood pressure. His body weight was 42 kg (20 kg less than before onset). Blood tests showed levels of hemoglobin: 90 g/L, white cells: 7.5 × 10^9^ /L with 81.8% band cells, platelets: 433 × 10^9^ /L, blood glucose level: 17.7 mmol/L, total iron binding capacity: 40.2 mol/L, transferrin: 147 mg/dL, prothrombin time: 14.2 s, prothrombin activity: 61.0 (80–150), alanine aminotransferase: 43 U/L, alkaline phosphatase: 394 U/L, and r-GT: 354 U/L. The tests for HIV Ab, anti-TB, EBV IgM, and syphilis were all negative. X-ray computed tomography (CT) showed left facial soft tissue deficiency, absence of left anterior bone wall of the maxillary sinus, mucosal thickening with increased density of bilateral maxillary sinus, ethmoid, sphenoid sinus, bilateral hydrothorax, and high-density fuzzy shadow in the lower part of the lungs. Meningeal enhancement was seen on MRI.

**Figure 2:**
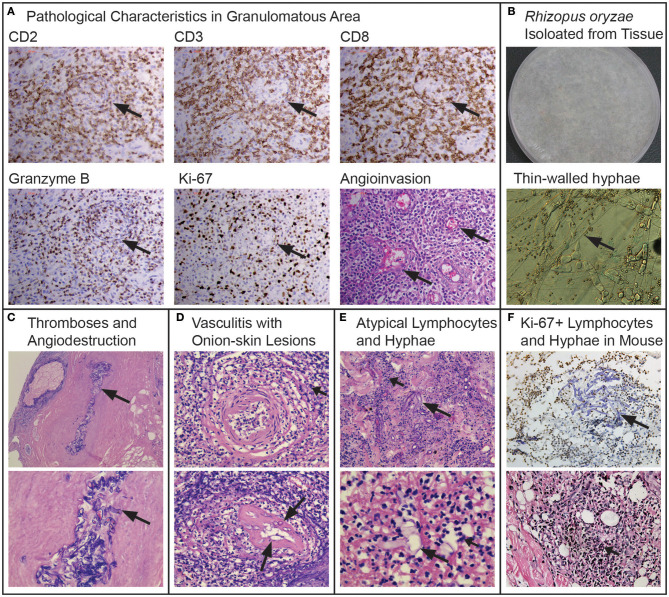
“*Rhizopus arrhizus* associated lethal midline granuloma/rhino-orbital cerebral mycosis.” Pathologic characteristics of the midline face destruction patient, the fungus and hyperplasia replicated in the mouse models. **(A)** Shows the pathological characteristics in the granulomatous area with immunohistochemically positive staining of CD2, CD3, CD8, GranzymeB, and Ki-67 from granulomatous tissues (Envision method, original magnification ×400) and angioinvasion (H&E, original magnification ×400). **(B)** *Rhizopus arrhizus* (PUTH 20111112) isolated from tissue: lanose appearance with grayish color 5 days growth on PDA and thin-walled hyphae (arrows) and conidia (lactophenol cotton blue staining, original magnification ×400). **(C)** Fungal thromboses (long arrow), angiodestruction and angiocentric coagulative necrosis (short arrows, H&E, original magnification ×200 and ×400, respectively). **(D)** Atypical cells (short arrows) and hyphae (long arrows) among Onion-skin lesions (H&E staining, original magnification ×400). **(E)** Atypical lymphoid cells (short arrows) and hyphae (long arrows) (H&E staining, original magnification ×400 and ×1,000, respectively). **(F)** Ki-67 positive cells and hyphae with immunochemical staining in mouse infected by *Rhizopus arrhizus* (PUTH 20111112) (Envision method, original magnification ×400) hyperplasia with H&E staining (original magnification ×400).

A skin biopsy was performed on the lesions of the nasal dorsum, nasal mucosa, and granuloma. The biopsied tissue was sent for lymphoma diagnosis to the authored pathologists for microbiological evaluation in our laboratory. Pathology showed inflammation, necrosis, and pleomorphic cell infiltration, with atypical hyperplasia. Angioinvasive, angiocentric, and angiodestructive characteristics were typical with “Onion-skin” lesions (Figure 2A). In immunohistochemistry, the atypical lymphoid cells were positive for NK/T cell markers (CD2+, CD3+, CD8+, and individual CD56+), cytotoxic granule associated proteins (GZMB and TIA1), and hyperplasia (Ki67+)—typical of ENKTL (Figure 2). Patient was diagnosed as LMG/T cell non-Hodgkin's lymphoma at the Department of Pathology, Peking University, according to the WHO classification of mature T- and NK-cell neoplasms with negative EBER and EBV testing (9, 10).

### Multi-Stage Diagnostics

Five biopsy specimens were taken from different tissues on the nasal mucosa, edges of the ulcers, and eschar were cultured and were positive for a fungus that was later identified as *Rhizopus* by morphology (Figure 2B), and as *R. arrhizus* (=*R. oryzae*) by rDNA sequencing (D1/D2 and ITS1-ITS2 regions). The isolates were sensitive to amphotericin B (AMB) and resistant to fluconazole, itraconazole, and voriconazole. Microscopy of pathological slides, which did not detect EBV RNA *EBER1* with *in situ* hybridization, revealed large amounts of broad, aseptate, thin-walled hyphae in the affected tissue, artery walls and lumens. Thrombosis was observed that was surrounded with ischemic necrosis. In addition, angiodestructive characteristics were seen along small arteries which were filled with hyphae. “Onion-skin” lesions with fungal elements were seen upon the cross section of the involved arteries (Figures 2C,D). Comparing multiple biopsy specimens sampled from the edge of the newly infected tissues with those in necrotic tissues, we found that typical hyphae could be observed in the newly affected tissues only, whereas atypical dysplasia were seen with positive T cell markers (a few spots with NK/T cell makers) in the granulomatous tissue, with thin-walled hyphal elements and spores that were broken or endocytosed by the nucleate or giant cells, some of which were atypical with a high index of Ki67 expression (Figure 2).

We re-diagnosed the patient as having LMG associated with *R. arrhizus* infection (ROCM/LMG) and initiated treatment with intravenous AMB (25 mg once daily) accordingly, and gradual improvement was observed as the ulcers became smaller. Thirty-four days after the onset of therapy, he was transferred to the Plastic Surgery Department for skin transplantation. AMB treatment was discontinued and a relapse was noted 12 days after. He returned to the local hospital and restarted AMB treatment. Because of the serious side effects of hypopotassemia and tachycardia, he was transferred to the Division of Nephrology in the General Air Force Hospital 1 week after the recurrence, where he was treated with liposomal AMB (L-AMB) at a dosage of 50 mg daily for 2 months. With a total dosage of 1,055 mg of AMB and 2,600 mg of L-AMB, the destructed area was completely healed with scars (Figure 1F). Ten months later, he was successfully transplanted with a thick skin graft from his thigh for the reconstruction of the nose and chin. At follow-up, he lives a normal life up to now.

## Methods

### Specimen Collection and Mycology

Five biopsy specimens taken from different tissues of the nasal mucosa, edges of ulcers, and eschar were cultured on Sabouraud's glucose agar (SGA) and yielded the same fungus recorded as PUTH 20111112. The organism recovered from the primary culture was grown on potato dextrose agar (PDA) at 28°C for 4 days. Genomic DNA of the isolate was extracted by using the Biospin Fungus Genomic DNA sequence of the internal transcribed spacer region (ITS) and the D1–D2 regions of the large subunit ribosomal DNA were amplified by using primer pairs ITS4/ITS5 (5′-GGTCCGTGTTTCAAGACGG-3′/5′-GGAAGTAAAAGTCGTAACAAGG-3′) and D1/D2 (5′- AGCGGAGGAAAAGAAACTA-3′/5′- ACGATCGATTTGCACGTCAG-3′), respectively. The amplification products were then sequenced by BGI (Beijing, China). The obtained sequences were compared with those in the GenBank (https://blast.ncbi.nlm.nih.gov/).

### Animal Experiments

#### Isolates and Mice

Subcultures of PUTH 20111112 were grown on SGA at 28°C for 4 days. Conidia were harvested by gently washing the surface of the slants with saline. The suspension was filtered through a sterile 40 μm cell strainer and the number of conidia was adjusted to 5 × 10^7^ colony-forming units per milliliter (CFU/ml) by counting the spores in a hemocytometer and subsequently verifying these results through quantitative colony counts on PDA plates. Resting conidia were immediately used or stored at +4°C.

Twenty ICR mice (male, 18–22 g, 6–8 weeks) were purchased from the SPF Animals Experimental Animal and Technology Co., Ltd. (Beijing) and bred in the animal experiment department of the 304 Hospital of PLA (Beijing) under specific-pathogen-free (SPF) conditions. Mice between 6 and 8 weeks of age were divided into two groups randomly, with 10 mice in each group. Group 1 was inoculated with spore suspension (5 McFarland standards) of *R. arrhizus* PUTH 20111112 on the back of the skin and group 2 with normal saline as the negative control.

#### Establishment of Skin Infection Models

Mice between 6 and 8 weeks of age were infected by an intracutaneous injection at the skin of the back with a suspension of *R*. *arrhizus*. Mice of group 1 were inoculated with *R*. *arrhizus* and mice of group 2 were intracutaneously injected with normal saline as control.

#### Access and Process of Skin Specimens

Every week after inoculation until week 5, skin specimens around the inoculation position from two mice in each group were collected to perform fungal culture in SDA and were embedded in paraffin and serially sectioned for the histopathological examination of Hematoxylin-eosin staining (H&E), Periodic acid-Schiff staining (PAS), Periodic Schiff-Methenamine staining (PASM), Calcofluor staining, and immunohistochemical staining.

#### Antibodies

The primary antibodies used for immunohistochemical staining targeting were CD56/NCAM (Neural Cell Adhesion Molecule), anti-CD3, PRF, GZMB, TIA1, and Ki-67 (all from Abcam, Boston, USA). The anti-rabbit antibody (Zhongshan Gold Bridge, Beijing, China) was used as the secondary antibody for the immunohistochemical staining. Unstained cells were used as negative controls and ovarian cancer slices were used as positive controls.

#### Immunohistochemical (IHC) Staining

Paraffin sections were dried at 60°C for 4 h, and the paraffin was removed successively by immersing them into dimethylbenzene substitutes (3 × 15 min), absolute ethanol (2 × 5 min), 95% ethanol (2 × 5 min), 80% ethanol (5 min), and distilled water (2 min). The samples were incubated with 3% peroxide-methanol in a light resistant container at room temperature for 10 min for endogenous peroxidase ablation. All following steps were carried out in a moist chamber. The samples were rinsed three times with clean water, three times with distilled water, and three times in PBS (phosphate buffer solution, pH 7.4). CD3, CD56, GZMB, TIA1, PRF, and Ki67 antigens were retrieved by a high pressure and high temperature antigen retrieval method using a pressure cooker and immersed in citric acid buffer (pH 6.0). The slides were then rinsed in PBS for 3 × 5 min. The anti-CD3, anti-CD56/NCAM, anti-granzyme B, anti-TIA, anti-PRF, and anti-Ki-67antibodies were diluted into 1:400–1:4,000 solutions separately and were dropped 50–100 μL onto the slides at 4°C overnight. Then, 50–100 μL secondary antibody liquid was dropped onto the slides and incubated for 30 min at room temperature. The slides were rinsed in PBS for another 3 × 5 min. All slides were colorized with 3,3-diaminobenzidin (DAB, Zhongshan Gold Bridge, Beijing, China) 40 s for Ki-67 coloration and 80 s for CD3, CD56, GZMB, TIA1, PRF, and CD56 coloration. The cell nuclei were counterstained with hematoxylin, and then the slides were dehydrated and integrated with neutral balsam.

## Results

### Mycology

Five biopsy specimens grew highly similar, grayish, filamentous colonies that were identified as *Rhizopus arrhizus* (*R. oryzae*) recorded as PUTH 20111112 (Peking University Third Hospital). With sequence analysis, the identification of *R. arrhizus* was confirmed with a high identity (≥99%), high query coverage (≥99%), and low E value (≤10^−5^).

### Animal Experiments

#### Skin Lesions Fungal Culture

One week after inoculation, the mouse skin tissues of group 1 (with fungus) were inflamed and swollen with subsequent ulcers, while the control mice (group 2) were noted as inflamed. Tissue re-cultivations for fungus were positive in group 1 and negative in group 2.

#### Pathological and Immunohistochemical Manifestations

**Figure 3:**
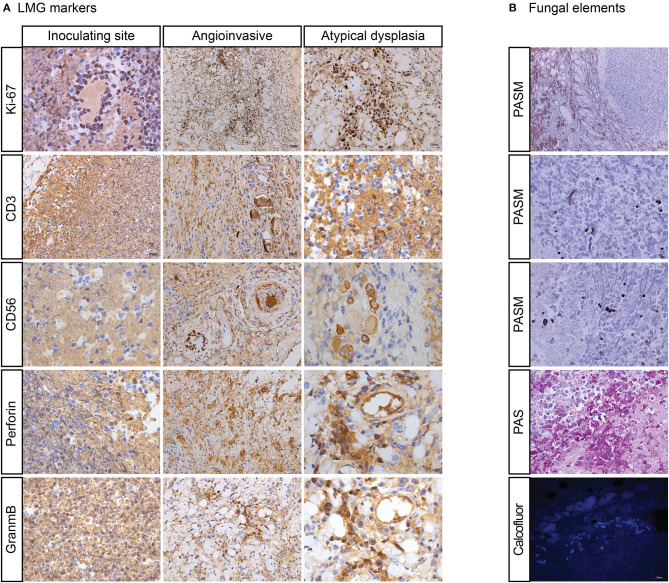
“Mouse infection modules with *Rhizopus arrhizus* isolated in LMG patient. **(A)** Shows atypical dysplasia with immunohistochemically positive staining of Ki-67, CD-3, CD-56, PRF, and GZMB. **(B)** Shows hyphae (broken and disintegrated) and spores (endocytosed) by periodic acid-silver metheramine (PASM), Periodic Acid-Schiff stain (PAS), and Calcofluor staining”.

Nodules and ulcers were induced by *R*. *arrhizus* PUTH 20111112. Under the microscope, angioinvasion, necrosis, granulation, and inflammation have been observed with pleomorphic cells. In some areas, atypical cells were seen with irregular nuclear margins and nuclear hyperchromasia. These cells were also positive for Ki-67, CD3, CD56, TIA1, GZMB, and PRF. Thin-walled hyphae and spores could be seen in the inoculated sites. The Ki-67 positive cells were mostly accumulated around hyphae (Figures 2F, 3). With an increased distance to the inoculation site, the mycelium became atypical and more difficult to identify. Thin-walled hyphae appeared in different forms and could be distorted, broken, disintegrated, or endocytosed that were hardly observed even with PAS and PASM staining (Figure 3).

## Discussions

Our patient was characterized by: (*a*) nasal airway obstruction with rhinorrhea and epistaxis; (*b*) progressive nose, palate, and middle face swelling, ulceration, and destruction with signs of orbital and cerebral involvement; (*c*) inflammation with pleomorphic cell infiltrations; (*d*) expression of NK-cell or T cell markers [CD2+, CD3+, CD56– (individual CD56+), CD8+], more than one cytotoxic granule associated proteins (GZMB, PRF, and T-cell restricted intracellular antigen); (*e*) vascular damage with angioinvasive, angiocentric, and angiodestructive lesions, and “Onion-skin” feature; (*f*) atypical dysplasia. The case was first diagnosed as LMG (T cell non-Hodgkin's lymphoma) (9, 10) and was expected to undergo chemotherapy after a multidisciplinary consultation. However, as *R. arrhizus* was detected, cultured, and examined with high loads in the arteries, we considered the T or NK cells to be secondary to the mucoralean infection, which might play a causal role in the development of the malignancy. We treated him with antifungal therapy rather than chemotherapy, and this led to a successful cure. Patient recovered completely with AMB and lives a normal life up to now. When inoculated to mice, *R. arrhizus* induced NK/T cell infiltration with cytotoxic granule associated proteins (CD3+, CD8+, CD56+, TIA1+, GZMB+, and PRF+) with hyperplasia (Ki67+) and vascular damage of angioinvasion similar to what was seen in the diseased patient.

LMG lists among the most malignant ulcers and may occur as a granulomatous response of microbial etiology (5–8). *Staphyloccus aureus* (30), *Nocardia farcinica* (31), *Mycobacterium fortuitum, M. marinum* (32), *Leishmania infantum* (33), and EBV (9–11, 34) are among the suspected causative agents. Mucoralean fungi have been reported to cause infections with highly similar clinical features (1, 24–26). Particularly, *M. irregularis* (28, 29) shows resemblance because this species causes chronic infection in patients without an apparent underlying disease, in contrast to other Mucoralean members which are usually acute in patients with leukemia or ketoacidotic diabetes. The present case of *R. arrhizus* infection exceptionally developed slowly, and the subsequent mouse infection experiments showed pathology characterized by hyperplasia and responsive natural killer cell or/and T cell infiltration with cytotoxic granule associated proteins, in addition to thin-walled hyphae in tissue and vascular damage.

*R. arrhizus* is the most common etiological agent of ROCM and is strongly associated with facial infection (20, 22, 35–37). Its prevalence is higher in Asia and South America compared to Europe and North America. Infections mostly involve elderly persons, and males are affected more frequently than females (38, 39). Most cases develop in patients with diabetes mellitus, commonly with a history of sinusitis or sometimes tooth extraction (38–40). Clinically, *R. arrhizus* associated ROCM is characterized by a progressive midline facial destruction, often with proptosis, ptosis, ophthalmoplegia, and symptoms of the brain (22, 38–40). Initial signs include nasal stuffiness, epistaxis, and facial swelling. With progression, lesions become necrotic with a purulent discharge. Necrosis of the sinuses, nose, face, orbit, and other midline destructions of the face can be seen that are also signs of LMG (22, 38, 39).

Angioinvasion is prominent in the pathogenesis of mucormycosis (41). Ibrahim et al. (42) examined the endothelial cell interactions of *R. arrhizus* and found that both alive and killed *R. arrhizus* hyphae were endocytosed by human umbilical vein endothelial cells. The endocytosed hyphae caused a significant endothelial cell damage, suggesting that a factor associated with the fungal cell wall is toxic to these cells. The damage to the endothelial cells resulted in the exposure of vascular smooth muscle cells, which can release large quantities of tissue factors and cause intravascular thrombosis (43). This may be viewed as a characteristic of mucormycosis and ROCM (22). In the present case, we found *R. arrhizus* elements in the artery lumens and walls, where thromboses with angioinvasive, angiocentric, and angiodestructive features suggested fungal damage to the arteries (Figures 2A,B).

*R. arrhizus*-induced NK/T cell infiltration (CD3+, CD56+, TIA1+, GZMB+, and PRF+) was seen in our patient and the fungus replicated in the mouse infection models. Typically in the LMG patient, T cell and NK cell infiltration with cytotoxic granule associated proteins (GZMB, PRF, and TIA1) was observed, providing one of the diagnostic criteria (9–12, 44–50). However, roles of these immune cells remain ambiguous with respect to a microbe-induced LMG syndrome (23, 28, 48). In the present *R. arrhizus*-associated midline face destruction and in previous *Mucor*-associated midline face destruction, T cells and/or NK cells with cytotoxic granule associated proteins (GZMB and TIA1) were observed. When introduced to mice, markers were re-expressed (Figures 2F, 3) (28, 29). Potenza et al. observed that Mucorales-specific T cells emerged in the course of infection in patients with invasive mucormycoses and that they exhibited direct antifungal activity comparable to that of either polymorphonuclear leukocytes or antigen presenting cells (51). Other experiments have shown that human NK cells or T cells could damage *R. arrhizus* and other fungi by PRF and GZMB-mediated apoptosis and by recruiting other immune cells to bind and then kill fungi (42, 52, 53). Recently, Deo et al. successfully incubated *in vitro* panfungal T cells to increase their number for clinical cell therapy with clinical *R. arrhizus* by the use of the blood and stem cells of healthy donors as the starting material (54). We consider this as an innate immune response, which might be an overreaction, mediated by cytotoxic CD8+ T and NK cells.

We observed atypical hyperplasia with a high Ki-67 expression in the patient and in mice with high loads of fungal elements. Significantly, these expressions were intensified in the cells around the fungal hypha with irregular nuclear margins and nuclear hyperchromasia positive for Ki-67, CD3, CD56, TIA1, GZMB, and PRF antigens with IHC staining. The phenomenon was also observed in our previous studies (28, 29). In 2012, we first reported the inducement of proliferation in a mid-face patient with *M. irregularis* infection (28). When introduced to mice, *M. irregularis* also induced a high Ki-67 expression, which was intensified around its hyphae (29). We considered the high Ki-67 expression around the fungi as activated NK and/or T cells in host defense against pathogens rather than as malignancy (55). Sample size is not sufficient for statistical analysis. However, our findings are suggestive for the role fungus plays in LMG.

In summary, we here describe a mid-face destruction patient, which was diagnosed by the top pathologists in China according to the current criteria of NK/T cell lymphoma, with a negative result for EBV and positive for *R. arrhizus*. With a then developed mouse experiment, the “malignant ulcer” in our case was associated with *R. arrhizus* infection, which was responsible for NK/T cell infiltration with cytotoxic granule associated proteins (CD3+, CD8+, CD56+, TIA1+, GZMB+, and PRF+), hyperplasia (Ki67+), and vascular damage of angioinvasion, suggesting another fungal etiological agent for LMG, which could be eradicated with AMB.

## Data Availability Statement

The original contributions presented in the study are included in the article/supplementary material, further inquiries can be directed to the corresponding author/s.

## Ethics Statement

The animal study was reviewed and approved by Peking University Third Hospita IRB; approval #00006761-2015025. Written informed consent has been obtained from the patient for publication of this case report.

## Author Contributions

DML and LDL diagnosed and treated the patient and wrote the manuscript. JG and GJZ did the mouse experiment. XLL assisted in the clinical administration. GSH identified the fungus and revised the manuscript. All authors contributed to the manuscript revision, read, and approved the submitted version.

## Conflict of Interest

The authors declare that the research was conducted in the absence of any commercial or financial relationships that could be construed as a potential conflict of interest.
